# Supplementary material for: ‘Thriving with bipolar disorder’: The co-design of a peer-delivered group psychoeducation program and single-arm pilot feasibility evaluation protocol
Source: PLoS One. 2025 Dec 17;20(12):e0338306. doi: 10.1371/journal.pone.0338306 (PMC12711008; doi:10.1371/journal.pone.0338306)
Supplement: S2 File — The full protocol and ethics approval from the University of British Columbia Behavioural Research Ethics board (H24-03489), including risk and data management processes. (PDF) [file pone.0338306.s002.pdf]

Date: 2025-07-02, 9:38:52 a.m.	Print	Close

undefined

The University of British Columbia
Office of Research Ethics
Behavioural Research Ethics Board
Suite 102, 6190 Agronomy Road
Vancouver, BC V6T 1Z3

_H24-03489_ Peer-led Bipolar Disorder Psychoeducation (Version 2.0)

Principal Investigator: Erin Michalak

1. Principal Investigator & Study Team - Human Ethics [View Form]

1.1. Principal Investigator

Last Name	First Name	Employer.Name	Email
Michalak	Erin	Psychiatry	erin.michalak@ubc.ca

Enter Principal Investigator's secondary appointments or affiliations (including Health Authorities), if applicable:

1.2. Primary Contact

Last Name	First Name	Rank
Poh	Tze Hern	M&P Staff

1.3A. Co-Investigators - Online Access

Last Name	First Name	Institution/ Department	Rank	TCPS2
Morton	Emma	Other/Other University/Hospital	Sessional Instructor/Lecturer	yes

1.3B. Describe each Co-I's role in study, e.g. statistician, supervisor, adviser, student etc. Ensure individual is entered in Box 1.3A

Dr. Erin Michalak and Dr. Emma Morton conceptualized and design this study.

Dr. Erin Michalak is a Professor in the Department of Psychiatry at the University of British Columbia in Vancouver, Canada. She is a world leader in consumer-involved psychosocial research in bipolar disorders (BD). Dr. Michalak founded and leads the Collaborative RESearch Team to study psychosocial factors in Bipolar Disorder (CREST.BD). CREST.BD's mandate is to advance research, clinical practice, and social change for psychosocial care and supports for BD through a community-based participatory research (CBPR) approach.

Dr. Michalak has co-developed the first disorder-specific quality of life measure for BD that will be used in this study. Her involvement will also draw on her extensive experience and demonstrable

	expertise in: quality of life and recovery assessment in bipolar disorder, psychosocial issues in bipolar disorder, community-based participatory research, knowledge translation and qualitative analyses. Dr. Michalak will oversee data collection, analysis and storage at UBC.<br><br>Dr. Emma Morton is a Senior Lecturer (Teaching and Research) in the School of Psychological Sciences at Monash University. She completed her PhD and clinical training as a psychologist at Swinburne University of Technology, Australia and her postdoctoral research fellowship at the University of British Columbia. Dr. Morton is now the co-director of the Collaborative Research Team to Study Psychosocial Issues in Bipolar Disorder (CREST.BD). Dr. Morton will oversee subject recruitment, data collection and will conduct analysis of the data.
1.4A. Additional Study Team Members - Online Access	see table below
1.4B. Describe each Additional Study Team Members' role in study, e.g. staff, research assistant etc.	Sahil Kanani is a research assistant and Brianna Hou is a WorkLearn student who will both support administrative activities associated with this study.
1.5A. Additional Study Team Members - No Online Access	see table below
1.5B. Describe each Additional Study Team Members' (no online access) role in study, e.g. external supervisor, consultant etc.	Andrew Kcomt is the Knowledge Translation & Research Manager at Hope + Me - Mood Disorders Association of Ontario. As the co-chair of the CAN-BIND Community Advisory Committee, he represents the community perspective on the Ontario Brain Institute’s Knowledge Translation, Outreach, and Communications team and has been a long-time community advisor with Collaborative RESearch Team to study psychosocial issues in Bipolar Disorder (CREST.BD).<br><br>Andrew Kcomt will help with recruitment and administering the psychoeducation program through Hope + Me, but will not have

1.4A. Additional Study Team Members - Online Access

Last Name	First Name	Institution/Department	Rank	TCPS2
Hou	Brianna	UBC/Medicine, Faculty of/ Psychiatry	Student Worker	yes
Kanani	Sahil	UBC/Medicine, Faculty of/ Psychiatry	M&P Staff	yes

1.5A. Additional Study Team Members - No Online Access

Last Name	First Name	Institution / Department	Rank / Job Title	Email Address
Kcomt	Andrew	Hope + Me (Mood Disorders Association of Ontario)	Research Consultant	research@mooddisorders.ca

	access to any research data. Andrew will also supervise program activities and will act as a liaison between Hope+Me and the research team at UBC.
1.6. Tri Council Policy Statement (TCPS) Tutorial<br><br>*Have all research personnel completed the required TCPS2 tutorial:*	Yes
1.7. Project Title<br><br>*Enter the title of this research study as it will appear on the certificate. Title given **must match** the title on all study documents.*	'Thriving with Bipolar Disorder': Co-design and Pilot Evaluation of a Peer-Delivered, Quality of Life Focused Group Psychoeducation Program
1.8. Project Nickname<br><br>*Enter a nickname for this study. What would you like this study to be known as to the Principal Investigator and study team?*	Peer-led Bipolar Disorder Psychoeducation
**2. Study Dates and Funding - Human Ethics** [View Form]	
You plan to start collecting data immediately after obtaining ethics and any other required approvals	yes
You plan to start data collection at a later date i.e., 2 months or more after approvals are obtained. Click the calendar icon below to select the dates.<br><br>*Estimated start date:*	
2.1.B.<br><br>*Estimated end date:*	2025-12-31
2.2.A. Types of Funds<br><br>*Please select the*	Grant

applicable box(es) below to indicate the type(s) of funding you are receiving to conduct this research. ***You must then complete section 2.3 and/or section 2.4 for the name of the source of the funds to be listed on the certificate of approval.***	
2.2.B. *For Industry Sponsored studies, please provide a sponsor contact.*	
2.3.A. *Research Funding Application/ Award Associated with the Study that was Submitted to the UBC Office of Research Ethics*	**UBC Number** / **Title** / **Sponsor**<br>F24-03795 / Pilot evaluation of a peer-delivered psychoeducation program for bipolar disorder / Laurel Foundation
2.3.B. *Which institution is administering the funds, if not UBC or UBC affiliated institution?*	
2.4.A. *Research Funding Application/ Award Associated with the Study not listed in question 2.3.*	**UBC Number** / **Title** / **Sponsor**
2.4.B. *Please enter any applicable information about your funding which is not already shown in Box 2.3A or 2.4A (including funding applied for but not yet received).*	This research was supported by a philanthropic donation from the Laurel Foundation. The funders have no influence on the design, conduct, or reporting of the study.
2.5.A. *Is this a DHHS grant?*	no
2.5.B. *Please select the appropriate DHHS funding agency from the selection box.*	

2.6. Study Related Conflict of Interest<br><br>*Conflicts of Interest (COIs) in research are situations where someone's personal interests (financial, career, or other) could compromise or could be perceived to compromise the objective conduct of research or integrity of the data. Conflicts of interest can arise naturally from an Investigator's engagement inside and outside the University, and the mere existence of a COI or the perception of a COI does not necessarily imply wrongdoing on anyone's part. Nonetheless, real and perceived COI must be recognized, disclosed, and assessed. This question asks Investigators to disclose COIs that may relate to the research study that is the subject of the REB application.*<br><br>*Do the Principal Investigator, Co-Investigators and/or their related parties have any personal interest(s) that could compromise or reasonably be perceived to compromise the objective conduct of the research or the integrity of the data generated by the study? Personal*	no

interests may include business, commercial or financial interests, dual roles (e.g. PI and Doctor), as well as personal matters and career interests.	
**4.A. Study Type - (Boxes 4.1 to 4.2C)** [View Form]	
4.1. Application Type<br><br>*Indicate whether your application is Clinical or Behavioural.*	Behavioural
4.2.A. *Institutions and Sites for Study (including study team members' institutional affiliations under which this research is being conducted)*	**Institution** **Site**<br>UBC Vancouver (excludes UBC Hospital)
4.2.B. *Non-UBC Institutions and Sites for Study (including study team members' institutional affiliations under which this research is being conducted)*	**Institution** **Site**
4.2.C. *Please enter any other locations where the research will be conducted under this Research Ethics Approval (e.g., Name of privately owned clinic, community centre, school, classroom, participant's home, in the field - provide details).*	The psychoeducation program will be delivered through the community organization, HOPE+Me (The Mood Disorders Association of Ontario). HOPE+Me is an Ontario-based charity that supports people living with mood and anxiety disorders through education, advocacy, training, and support services. They have one of the largest community infrastructures within the national network of mood disorders associations, serving over 76,000 people province-wide. HOPE+Me also provides services tailored to the needs of marginalized and racialized communities, such as dedicated LGBTQ+ and Black, Indigenous, and People of Colour peer support groups. Hope+Me specializes in virtual peer support (see: https://hopeandme.org/pathways-to-care/virtual-peer-support-groups/) and has standard operating procedures for virtual engagement.<br><br>Peer facilitators will be recruited through HOPE+Me, with ongoing supervision provided by HOPE+Me and CREST.BD, jointly. Program participants will be recruited through HOPE+Me and CREST.BD channels. All study data will be collected using UBC-based tools such as Qualtrics, and stored at UBC-Vancouver during

	this study. Interviews will take place using online, or telephone-based methods of contact through UBC.

4.B. Behavioural Study Type - (Boxes 4.2D to 4.6) [View Form]

	Study Site:	Accessing Records or Charts:	Analysing Data or Utilizing Lab Space:	Recruiting Participants:	Team Member Affiliations:
4.2.D. Roles of Study Sites and Institutions	UBC - Vancouver (excludes UBC Hospital)	yes	yes	yes	yes

4.3.A. *If this proposal is closely linked to any other proposal previously/ simultaneously submitted, enter the Institution or Health Authority name and associated Research Ethics Board study number of that proposal.*<br><br>*Institution Name:*	
REB study number:	H23-01462
4.3.B. *If applicable, please describe the relationship between this proposal and the previously/ simultaneously submitted proposal listed above.*	CREST.BD has successfully utilized digital platforms (e.g., the Polarus App) for self-management knowledge translation (KT). While digital tools represent one promising avenue for BD care, they do not address the needs of all individuals, such as those impacted by the digital divide or facing additional barriers such as physical disability. To support equitable and inclusive access to this information, diverse avenues of delivery are required. One candidate alternative delivery format is face-to-face, peer delivered self-management psychoeducation and support.<br><br>In H23-01462, we conducted focus groups to inform the creation of a peer-delivered psychoeducation program. Manuals for facilitators and attendees have now been created, drawing on the knowledge base integrated in the PolarUs app.<br><br>This ethics application corresponds to the administration and a pilot evaluation of the newly developed, peer-delivered psychoeducation program.
4.3.C. *Have you received any information or are you aware of any*	no

rejection of this study by any Research Ethics Board? If yes, please provide known details and attach any available relevant documentation in Box 9.7.	
Please provide known details:	
***4.4.A.** External peer review details:*	
***4.4.B.** Internal (Institution or hospital) peer review details:*	
***4.4.C.** If this research proposal has not received any independent scientific/ methodological peer review, explain why no review has taken place.*	
Participant Vulnerability	Medium
Research Risk	Medium
***4.5.B.** Provide explanations for the assessments of research risk and participant vulnerability reported above.*	Participant Vulnerability:<br><br>Participant vulnerability in the psychoeducation program, and subsequent pilot evaluation, is estimated to be medium given the potential for participants to experience an episode that could impact their capacity to consent and/or participate in the program and evaluation. Participants will be reminded of their right to withdraw their participation at any time before and during the program and/or evaluation. Additional details are specified in section 6.9.<br><br>If there is any indication that a study participant may be in severe distress or at present risk of self-harm or suicide during the program, a Hope+Me staff member will be available to provide one-on-one support. Additionally, provided emergency contacts will be utilized, if required. If one-on-one support was needed by a participant, a case note (as per the attached template 'Drop-In Support Case Notes') will be completed documenting the participant's primary concern, strengths and supports, personal goals and coping strategies. Facilitators and the one-on-one support staff member will have access to a number of resources, including coping skills, grounding techniques, along with access to crisis services and virtual urgent care. In case of any similar events during research activities (e.g., in email correspondence from participants, during qualitative interviews), a 'Risk Management and Distress Protocol' (attached) has been developed for research staff

	to utilize.<br><br>To protect confidentiality, participants in the psychoeducation program will be reminded to not share material discussed with people who did not attend. Attendees in the program will be asked to identify themselves by first name/pseudonym only to maintain anonymity.<br><br>Participation in all research activities is expected to have no direct effect on the rights, opportunities, privileges, or power that participants already hold or are entitled to.<br><br>Research Risk:<br><br>Research risk in the psychoeducation program is estimated to be medium. Participants will talk about mental health and self-management practices, and questions might provoke mild psychological discomfort; such effects would be expected to be transient. Program facilitators will be trained on guiding conversations and managing group dynamics (see section 6.2 for details of the training facilitators will have access to), making the probability of such a risk minimal. A list of mental health resources useful for participants during and beyond the program will also be provided to participants.<br><br>Research risk in the pilot evaluation component is estimated as low. Participants will be asked to divulge limited personal information in audio-recorded interviews, transcripts will be de-identified (names removed) and any identifying information will be stored by the investigators in records separate from study data.
4.5.C. *Does your application fall under minimal risk (i.e., was it assigned an overall risk level of 1 or a blue box on the minimal risk matrix above)?*	no
**4.C. Behavioural Study Type - (Boxes 4.7 to 4.8)** [View Form]	
4.7.A Creation of a Research Database or Registry<br><br>*Does this study involve the creation of a research database or registry with a local custodian for future unspecified research?*	no

4.7.B. *Is the purpose of this application exclusively to obtain approval for the creation of a research database or registry? [Note: if the creation of the database or registry is part of a bigger project also included in this application, you must answer "no" below].*	no
4.8. Course-based research project<br><br>*Please review the guidance on submitting course-based research projects before responding, to confirm that your application will meet the criteria.*<br><br>*Is this application intended to cover projects conducted for pedagogical purposes within a course?*	no
Survey Research<br><br>*Is this a **minimal risk** study exclusively using a survey for data collection?*	no
Secondary Use<br><br>*Is this a **minimal risk** study exclusively analyzing previously collected data?*	
**5. Summary of Study and Recruitment - Behavioural Study** [View Form]	
5.1.A. *Provide a brief statement about the project written in lay language. Do not exceed 100 words and do not cut and paste directly from the study*	Self-management strategies can be used by individuals with bipolar disorder (BD) to cope with symptoms and improve quality of life (QoL). Peer-facilitated education programs have the potential to diversify delivery of self-management information by capitalizing on the expertise of individuals who live well with BD. We have co-designed a novel, peer-facilitated, QoL-focused, group education program for people living with BD. This project will involve

proposal.	administration of the program and a pilot evaluation of the feasibility, acceptability, and efficacy of this program for self-management of BD.
5.1.B. *Summarize the research proposal, including study purpose, hypothesis, study population, and research method.*	Background and Purpose:<br><br>Peer support (where individuals with shared lived experience of a mental health condition provide each other with informational, emotional, and social support) may be an acceptable way to disseminate information on self-management strategies, capitalizing on the expertise and knowledge of people who live well with BD.<br><br>Peer-facilitated group psychoeducation includes the benefits of evidence-based self-management information and tools, with the added advantage of providing role models for recovery and modelling of self-management skills (Proudfoot et al., 2012) (Tse et al., 2019). Unfortunately, there is a dearth of BD-specific peer-facilitated self-management psychoeducation programs, and corresponding evaluations of their efficacy.<br><br>To capitalize on the potential of peer support to enhance the delivery of BD self-management information, content from two, web-based, self-directed psychoeducational interventions was adapted to create a peer-facilitated psychoeducation program using a community-based participatory research (CBPR) framework. The resulting program contains eight, weekly, two-hour sessions. Each session focuses on a topic related to QoL in BD (including Mood, Sleep, Physical Health, Relationships, Money, Self-esteem, and Independence), and contains a combination of education, opportunities for peer-to-peer knowledge exchange, and activities that facilitate practice of self-management strategies. A facilitator and attendee manual have been created for use in this program.<br><br>Methods:<br><br>The project will be implemented across two phases:<br><br>In the first phase, which we have already completed, we have applied CBPR principles to develop a peer-facilitated, QoL-focused group psychoeducation program for individuals with BD. The resulting program, entitled 'Thriving with bipolar disorder' will be delivered in eight, weekly, two-hour sessions that will be co-facilitated by two peer facilitators with lived experience of BD.<br><br>In the second phase, we will conduct a pilot evaluation of the feasibility, acceptability, and preliminary efficacy of the program. The program will be delivered through Hope+Me, an Ontario-based charity that supports people living with mood and anxiety disorders through education, advocacy, training, and support services. Four peer facilitators will be recruited through Hope+Me and will attend a

	~5.5 hour training session prior to commencing the program. We will aim to recruit 32-40 program participants (8-10 per group) given previous research summarizing the ideal group size (Biggs et al., 2020).<br><br>The evaluation will be conducted as a single-arm, uncontrolled, pilot feasibility trial. An explanatory sequential mixed-methods design will be used (Creswell & Clark, 2011). The following data will be collected:<br><br>- Participants will provide baseline demographic and clinical information in a Qualtrics survey as part of their consent.<br>- Participants will complete Qualtrics surveys for efficacy measures at baseline, immediately after completing the program and 1 month after completing the program.<br>- Program feedback will be collected from participants immediately after completing the program through a Qualtrics survey.<br>- Peer facilitators will complete a brief, post-session Qualtrics survey each week, recording attendance, fidelity, and session feedback.<br><br>At the end of the intervention period, a subset of consenting participants (~n=12) and peer facilitators (~n=4) will be invited to participate in a one-hour qualitative individual interview over Zoom.<br><br>Study Population:<br><br>Four peer facilitators will be recruited through Hope+Me. Inclusion criteria for program participants are: (1) aged 18 or older, (2) a self-reported diagnosis of a mood disorder, (3) sufficient access to an internet-enabled computer or smartphone device through which they can access the Zoom teleconferencing platform, and (4) ability to understand, read and write English, (5) at least one year of prior experience leading peer support groups or recovery programs through Hope+Me, and have previously received training through Hope+Me and (6) have completed a Criminal Record Check (https://rcmp.ca/en/criminal-records/criminal-record-checks).<br><br>Inclusion criteria for program participants are: (1) aged 18 or older, (2) residing in Canada, (3) a self-reported diagnosis of BD, (4) ability to understand, read and write English and (5) sufficient access to an internet-enabled computer or smartphone device to access the Zoom teleconferencing platform. Exclusion criterion will be the inability to communicate in written and verbal English to a sufficient level to allow participation in the program and research activities.
5.2. Inclusion Criteria<br><br>*Describe the participants*	Four peer facilitators will be recruited through Hope+Me. Inclusion criteria for program facilitators are: (1) aged 18 or older, (2) a self-reported diagnosis of a mood disorder, (3) sufficient access to an

being selected for this study, and list the criteria for their inclusion.	internet-enabled computer or smartphone device through which they can access the Zoom teleconferencing platform, (4) ability to understand, read and write English, (5) at least one year of prior experience leading peer support groups or recovery programs through Hope+Me, and have previously received training through Hope+Me and (6) have completed a Criminal Record Check (https://rcmp.ca/en/criminal-records/criminal-record-checks).<br><br>Inclusion criteria for program participants are: (1) aged 18 or older, (2) residing in Canada, (3) a self-reported diagnosis of BD, (4) ability to understand, read and write English and (5) sufficient access to an internet-enabled computer or smartphone device.
5.3. Exclusion Criteria<br><br>*Include details if otherwise eligible participants will be excluded due to other characteristics. If no exclusion criteria are applicable, enter n/a.*	Exclusion criterion for both facilitators and participants is the inability to communicate in written and verbal English to a sufficient level to allow participation in the program and research activities.
5.4. Recruitment<br><br>*Provide a detailed description of the steps you will use to recruit participants. Include:*<br>*a) How will prospective participants be identified?*<br>*b) By what means will recruitment be done (e.g., public posting, direct contact, third party recruitment, etc.)?*<br>*c) Who will contact prospective participants?*<br>*d) If recruitment will occur in person, what sites will be used (e.g. doctor's office, hospital clinic, etc.)?*<br>*e) Attach all materials, including letters of initial contact, posters, scripts and advertisements, to Box 9.4.*	All recruitment for this study will occur online. Relevant recruitment materials have been attached in the additional documentation section.<br><br>Peer facilitators will be recruited through Hope+Me by promotion via the Hope+Me newsletter and existing peer support programs as per the inclusion criteria stated in 5.2. Interested peer facilitators will review the explanatory statement and provide informed consent using Qualtrics, prior to any intervention training.<br><br>Program participant recruitment will occur through promotion via the Hope+Me newsletter and existing peer support programs, as well as notices on CREST.BD social media pages, paid advertisements on Facebook, Instagram, and Twitter, emails to the CREST.BD mailing list, and Canadian healthcare providers or organizations associated with the CREST.BD network.<br><br>Interested participants will be provided a link to a Qualtrics survey where they will be able to review the explanatory statement, register their consent to participate, confirm their eligibility, and provide baseline demographic and clinical information.<br><br>Following registration, participants will register for the program through Hope+Me, confirm the suitability of the timing of sessions and review participation requirements.

5.5. Use of Records<br><br>*If existing records (e.g., health records, course grade sheets or other records/databases) will be used to access information about potential participants, please describe how permission to access this information, and to collect and use this information, will be obtained.*	N/A
5.6. Summary of Procedures<br><br>*Describe briefly in a step-by-step manner what the researcher will be doing with participants, after they have been recruited and consented.*	Facilitators will attend one ~5.5 hour training session provided by Dr. Erin Michalak to review the program structure, delivery, session-by-session objectives, and use of the facilitator manual. This training will also cover relevant components of the TCPS2 that extend to the facilitator role, including acquiring renewed consent, managing conflicts of interest, and maintaining privacy and confidentiality.<br><br>Prior and ongoing training and supervision through Hope+Me will allow facilitators to experience an extensive training model with programs such as safeTALK, de-escalation technique training, WRAP® training, ongoing monthly Community of Practice meetings, peer debriefing channels following sessions, and support from experienced peer support group facilitators. Details about these programs are provided in section 6.2 and in the attached documentation titled 'Peer Facilitator Guidelines and Supports'.<br><br>The program will be delivered virtually using Zoom teleconferencing software. Participants will be sent calendar invites containing the Zoom links for each session, and instructions on how to download and use Zoom. In the week prior to the first session, participants will be provided with a copy of the attendee manual and a link to a Qualtrics form where they will complete baseline efficacy measures.<br><br>We aim to recruit 32-40 program participants, assigning 8-10 participants per group (for a total of 4 groups). Each group will be co-facilitated by two peer facilitators with lived experience of BD. Each group will attend eight, two-hour sessions each week. All eight sessions follow the same general format: Participants will first enter a welcome area, where they will be introduced to their two group facilitators and another Hope+Me staff member that is on standby, should a client require one-on-one support. The document ‘Guidelines for Virtual Groups’ (attached in documentation) will be reviewed prior to the group starting. Peer facilitators will present an

	overview of the session outline, before facilitating a brief review of the participants' experiences of the previous week's take-home activities. The peer facilitators will then provide psychoeducation on the week's QoL topic and specific self-management strategies, and facilitate group discussions to reinforce specific concepts and provide opportunities for peer support and learning. They will also guide completion of structured skills practice activities, designed to support participants to understand and apply the self-management strategies. After each group, facilitators debrief and complete a 'Group Peer Support Summary' (attached in documentation) that captures the number of participants, how many were engaged, gender pronouns used, topics discussed, details if a conflict occurred. If one-on-one support was needed, a case note (as per the attached template 'Drop-In Support Case Notes') is completed documenting the participant's primary concern, strengths and supports, personal goals and coping strategies. Facilitators and the one-on-one support staff member have access to a number of resources including coping skills, grounding techniques, along with access to crisis services and virtual urgent care contacts, if needed.<br><br>Immediately post-program completion, participants will be provided with a link to a Qualtrics form to provide program feedback and complete efficacy measures. Participants will be recontacted after 1 month to complete final, follow-up efficacy measures.<br><br>Peer facilitators will complete a brief post-session Qualtrics survey each week, recording attendance, fidelity, and session feedback ('Weekly Facilitator Feedback' in documentation). Additionally, facilitators will debrief and complete a Hope+Me 'Group Peer Support Summary' document that captures the number of participants, how many were engaged, gender pronouns used, topics discussed, details if a conflict occurred.<br><br>At the end of the intervention period, a subset of consenting participants (~n=12) and peer facilitators (~n=4) will be invited to participate in an (optional) one-hour qualitative individual interview. All interviews will be conducted remotely via Zoom and will be recorded and transcribed for later analysis.
5.7. Research Types<br><br>*Select all that apply to your study. Please review the research methods descriptions before responding. If none apply, please select "None of these Methods"*	Community Based Research (collaboration with community on design and methods)

6. Participant Information and Consent Process - Behavioural Study [View Form]

6.1. Time to Participate	Each facilitator will run two psychoeducation groups. The number of hours has been scaled to account for this:<br><br>- one training session prior to program commencement (5.5 hours)<br>- session preparation: one hour per session, for 8 sessions/group (1 hour x 16 sessions) = 16 hours<br>- facilitation: eight, weekly, two-hour sessions per group (2 hours x 16 sessions = 32 hours)<br>- data collection: time associated with completing brief, post-session Qualtrics survey and group peer support summary each week for 8 sessions/group (0.25 hours x 16 sessions = 4 hours)<br>- (optional) qualitative individual interview at the end of the program (1 hour)<br><br>For participants:<br><br>- baseline efficacy measures survey (0.5 hours)<br>- program attendance: eight, weekly, two-hour sessions (2 hours x 8 = 16 hours)<br>- program feedback and efficacy measures survey after program completion (0.5 hours)<br>- one-month followup efficacy measures survey (0.5 hours)<br>- (optional) qualitative individual interview at the end of the program (1 hour)
6.2. Risks and Mitigation	Peer facilitators eligible for this program will have already received a minimum of 300 hours of training from Hope+Me and a minimum of 1 year of prior experience with facilitating a peer support group. This will be essential for providing support to others and ensuring facilitators can effectively manage challenging situations. Prior and ongoing facilitator training will include Hope+Me programs like safeTALK, de-escalation techniques, WRAP® training, and ongoing monthly Community of Practice meetings that includes review of topics such as conflict resolution. Peer facilitators also shadow a trained facilitator when first starting.<br><br>We have attached a document titled 'Peer Facilitator Guidelines and Supports' that clarifies (1) training and supports for program facilitators and (2) management of risks for both facilitators and attendees during the program. The document references 4 additional documents that further detail: (1) reflective practices for facilitators to self-assess their competencies, (2) guidelines for virtual engagement, (3) facilitator evaluations of each session, and (4) how peer facilitators can ask for support during the course of program activities.<br><br>Some of the training and supports available to peer facilitators in this program will include:<br><br>- To manage risk within Hope+Me support groups, all peer

facilitators and clients have access to a counselor and/or staff member during support groups. Facilitators will also have access to ongoing supervision and support before and after facilitating groups from these individuals.

- Hope+Me peer facilitators attend monthly community of practice (COP) meetings with other peer facilitators. These meetings include discussion of strategies to deal with conflicts in group settings, how to provide one-on-one peer support, and the Behavioural Influence Stairway Model. COP meetings also teach peer facilitators to recognize and address conflicts.

- Group facilitators also provide each other with peer supervision when co-facilitating sessions. Prior to each session, both peer facilitators will complete the ‘Expressing my needs and requesting support’ reflective exercise (referenced in the ‘Peer Facilitator Guidelines and Support’ document attached) which encourages them to reflect on their own wellbeing and emotional state, how they can meet their own needs with self-care, other self-management, or seeking support, and what support their fellow peer facilitator can provide during and after the session. Following group sessions, peer facilitators will engage in debriefing with co-facilitators and Hope+Me supervisors.

- Hope+Me specializes in virtual peer support (see: https://hopeandme.org/pathways-to-care/virtual-peer-support-groups/) so all facilitators will be sufficiently trained for virtual engagement. Additionally, group guidelines outline the boundary expectations that all participants agree too. If behavioural issues emerge during the session, participants can be placed into a breakout room to talk to a counsellor or peer support worker. Hope+Me also advocates for self-determination, allowing participants to freely enter a breakout room if they feel triggered or need one-on-one support.

- If a participant experiences increased emotional stress as a result of their participation, or may be in distress, facilitators have been trained to identify participants in need of support and decide how to proceed (e.g., to encourage use of support services, to access one-on-one support from a Hope+Me staff member, or to contact the attendee’s emergency contact). If one-on-one support was needed by a participant, a case note (as per the attached template ‘Drop-In Support Case Notes’) will be completed documenting the participant’s primary concern, strengths and supports, personal goals and coping strategies. Facilitators and the one-on-one support staff member have access to a number of resources, including coping skills, grounding techniques, along with access to crisis services and virtual urgent care contacts. In case of any similar events during research activities (e.g., in email correspondence from participants, during qualitative interviews), a ‘Risk Management and Distress Protocol’ (attached) has been

developed for research staff to utilize.

- To ensure the wellbeing of participants, All Hope + Me staff and volunteers are required to do a criminal record check (https://rcmp.ca/en/criminal-records/criminal-record-checks) before they can work with clients. Participants will be asked to agree to group guidelines, ensuring a productive and cohesive atmosphere and clear boundaries for all participants. Hope + Me staff and counsellors are also available on standby should any participant require additional support.

Some of the considerations to mitigate risks for participants in this program include:

- Questions and discussions about mental health might provoke mild psychological distress for people living with mood disorders, but these would not be expected to persist beyond the duration of study-related activities.

- We understand that our research participants who live with a mood disorder require special considerations in terms of power differentials between research participants, facilitators and researchers. With this in mind, participation will not be open to individuals under 18 years old. Similarly, facilitators will receive training on practices to foster diversity and inclusion in their groups.

- We will remind participants of their right not to take part in any aspect of the program or evaluation, and their ability to withdraw participation at any time, without providing a reason.

- When accessing a support group, attendees first enter a welcome area, where they are introduced to two group facilitators and another Hope+Me staff member (who has completed the same risk management and de-escalation training as peer facilitators) that is on standby, should a client require one on one support.

- Prior to joining a support group, Hope+Me provides attendees with an online form that requires their contact information, emergency contact information, and a Waiver and Release of Liability for Virtual Support, that must be completed prior to accessing a support group. As this is a real-world feasibility study, participants will provide this information to Hope+Me in line with their usual processes.

- A list of appropriate and available help resources will be provided to each participant in this study as part of their attendee manual (see attached documentation). Participants will also be supported in Session 1 to populate their own list of emergency contacts, including support persons, healthcare providers, and preferred crisis support resources, in order to ensure that their support options are also tailored to their preferences. This will especially be relevant for

	participants who express distress from discussions around depression/mania.<br><br>- All participants will be encouraged to contact the research team if they have questions or concerns about the evaluation components of the program. Participants with questions about the program will be able to contact an on-call Hope+Me staff member (including counsellors, social workers, and other staff who have completed the appropriate risk management and de-escalation training) during business operating hours. Andrew Kcomt from Hope+Me, will also be available as a liaison between program and research activities
6.3. Potential Benefits	Participants might or might not benefit from this project. Participants will gain access to a free 8-week psychoeducation program with tailored resources and activities on self-management strategies to optimize QoL. Participating in group workshop discussions will also give participants an opportunity to discuss their experiences, meet other people living with BD in their community, and make social connections. Because this program will be run as a pilot evaluation for a research study, participating in this program may generate positive altruistic feelings from contributing to the advancement of peer support programs specific to BD.
6.4. Impacts on Community	This study will address limitations in existing peer-support interventions, specifically, the lack of a BD-specific framework. Research with individuals with psychosis suggests that the success of a peer-delivered intervention may be impacted by the degree to which participants can relate to their peers as role models, as well as their use of self-management and coping strategies (Williams et al., 2018). Given this, a program that is BD-specific (rather than inclusive of mental illnesses more broadly) may be particularly beneficial.<br><br>As peer support interventions are increasingly embedded in mental health policy worldwide, we anticipate this project to have benefits for individuals living with BD in Canada and beyond. We expect this project to advance knowledge of effective peer support models, and contribute to the advancement of a lived experience workforce with specific understanding of BD-related needs and treatments.
6.5. Reimbursement and Incentives	Peer facilitators will be provided an honorarium of $50 CAD per hour for their participation in the pilot evaluation, including time associated with training, program delivery, and data collection.<br><br>Participants will be paid for completing the evaluation surveys during the program and, optionally, for participating in the follow-up qualitative interview at the conclusion of the study. Participants will be offered a $30 CAD Visa gift card for completing each survey, for a maximum of $90 CAD in Visa gift cards per participant. Participants who choose to participate in the optional, one-hour follow-up interview will be provided with an additional $30 CAD Visa gift card. Participants who choose to withdraw partway through a

	survey, interview or the program will still be offered the honorarium for study activities they participated in.<br><br>As this program and all corresponding research study activities will take place using remote contact methods (online, telephone) no reimbursements for travel costs will be offered.
6.6. Obtaining Consent<br><br>*Include details of where and when consent will be obtained and how it will be documented.*	Interested participants will be provided a link to the online survey platform, Qualtrics, where they will be able to review the explanatory statement, register their consent to participate, confirm their eligibility, and provide baseline demographic and clinical information. Following registration, participants will be contacted by Hope+Me to confirm the suitability of the timing of sessions and review participation requirements.<br><br>Interested facilitators will be sent a link to the online survey platform, Qualtrics, where they can review the explanatory statement, register their consent to participate, confirm their eligibility, and provide demographic and clinical information.<br><br>If there are any questions regarding the consent form or the study, contacts of the research staff will be provided. Research staff will be available to explain the consent process verbally, if requested, and answer any questions potential participants may have.<br><br>Verbal consent will also be obtained immediately before proceeding with/recording the optional qualitative interview at the end of the program.<br><br>Participants and facilitators will be reminded that they have a right to withdraw participation at any time before completion of the program or the evaluation components, without providing a reason. Details about this have been specified in Section 6.9.<br><br>Regarding the use of Zoom for program sessions, participants and facilitators will be reminded that UBC Zoom may collect data (usernames, email addresses). Participants will have the option of turning their camera on or off during the session, and can identify themselves through a first name or pseudonym if they choose.<br><br>Participants and facilitators will be encouraged to contact us if they have questions or concerns about the study or the question content that they would like to discuss further.
6.6.A. Waiver of Consent	N/A
6.7. Time to Decide	Participants and facilitators will be given a minimum of 7 days to decide whether they choose to participate in the program or evaluation components, as well as any additional time needed/ requested on an individual basis.

6.8. Capacity to Consent<br><br>*Will participants have the capacity to give fully informed consent on their own behalf?*	Yes
6.8.A. *Provide details of the nature of the incapacity (for instance, young age, mental or physical condition).*	
6.8.B. *If a participant does not have the capacity to give fully informed consent, who will consent on their behalf? Ensure the relevant consent form (parent/caregiver, substitute decision maker, legally authorized representative) is attached to page 9.*	
6.8.C. *If a participant does not have the capacity to give fully informed consent, will they be able to give assent to participate?*	
6.8.D. *If yes, explain how assent will be sought. Please be sure to attach copies of the assent form to page 9.*	
6.9. Ongoing Consent	We acknowledge that some participants may be at risk of making an impulsive consent decision at the outset of the study, or may change their mind about participating during the course of the program. Participants will be reminded of their right to withdraw their participation from the program at any time in the following ways:<br><br>- Within emails sent to remind program participants of an upcoming program session<br><br>- At the beginning of each session, in a ‘housekeeping slide’, and when the ‘Guidelines for Engagement’ document is reviewed.<br><br>- After each session, in a post-workshop email providing information

	about the next scheduled workshop.<br><br>The following text will be included in these communications:<br><br>"We would like to remind you of your right to withdraw from program activities by not attending or leaving a session at any time. If you withdraw from program activities, you can still complete the study evaluations and will be entitled to receive the honorarium provided. If you would like to withdraw from the study entirely, you may do so by contacting the study research team through email at crest.bd@ubc.ca"<br><br>A more formal re-consent process has also been embedded into relevant study surveys (post-program and follow-up surveys for attendees, and the weekly facilitator feedback survey) and the (optional) end-of-program qualitative interview. Participants will also be asked to re-consent mid-way through the program (during session 4).
6.10. Provisions for Consent (e.g., special assistance, Braille, translations/translator)	Those who require additional support understanding the consent forms may request to discuss the forms either over the phone or via email with the research team.<br><br>Unfortunately, it is not in our study budget to pay for translation and interpretation of forms, questionnaires, manuals and program delivery. Given the nature of small group participation, and to protect the confidentiality of other attendees, we will unfortunately not be able to accommodate use of volunteers/friends/family to provide interpretation for non-English speakers.
6.11. Restrictions on Disclosure	No restrictions have been placed on the disclosure of results.
**7. Number of Participants - Behavioural Study** [View Form]	
7.1. External Approvals<br><br>***A.*** *Other Institutions:*	no
B. *Please select "Add" to enter the name of the institution and attach the approval letter if received.*	**Name of Institution**
C. *Other Jurisdiction or Country (if "NO," go to 7.1.G):*	no

D. *Please select "Add" to enter the name of the jurisdiction or country and if you have already received approval attach the approval letter.*	**Name of Jurisdiction or Country**
E. *Has a Request for Ethics Approval been submitted to the institution or responsible authority in the other jurisdiction or country? (**Append a copy of any such document to this application once it is received**).*	no
F. *If a Request for Approval has **not been** submitted, provide the reasons below:*	N/A
G. *Does this research focus on Indigenous peoples, communities or organizations?*	no
G.1.A. *Will the research be conducted on Indigenous reserves, Métis settlement(s), or lands governed under a self-government agreement or an Inuit or First Nations land claims agreement?*	
If yes, please provide details:	
G.1.B. *Do any of the criteria for participation include membership in an Indigenous community, group of communities, or organization, including urban Indigenous populations?*	
If yes, please provide details:	

G.1.C. *Does the research seek input from participants regarding a community's cultural heritage, artifacts, traditional knowledge or unique characteristics?*	
If yes, please provide details:	
G.1.D. *Will Indigenous identity or membership in an Indigenous community be used as a variable for the purposes of analysis?*	
If yes, please provide details:	
G.1.E. *Will the results of the research refer to Indigenous communities, peoples, language, history or culture?*	
If yes, please provide details:	
G.2. Community Engagement<br><br>***G.2.A.*** *If you answered yes to questions a), b), c), d), or e), have you initiated or do you intend to initiate an engagement process with the Indigenous collective, community or communities for this study?*	
G.2.B. *If you answered "Yes" to question G.2.A., describe the process that you have followed or will follow with respect to community engagement. Include the role or position of those consulted, including their names if appropriate.*	

Attach any documentation of consultations (i.e. formal research agreement, letter of approval, email communications, etc.) below.				
Attachment:				
G.3. No community consultation or engagement<br><br>*If you answered "no" to question G.2.A., briefly describe why community engagement will not be sought and how you can conduct a study that respects Indigenous communities and participants in the absence of community engagement.*				
H. *Registration for Publication of Clinical Trials.*	no			
If 'Yes', click 'Add' to enter the following information.	**Has it been registered?**	**Indicate the Authorized Registry used:**	**Enter your Clinical Trial unique identifier:**	
7.2. Number of Participants<br><br>***A.*** *How many participants will take part in the entire study (i.e., world-wide)?*	44			
B. *How many participants will take part at institutions covered by this Research Ethics Approval?*	44			
7.3. Principal Investigator and Research Team Experience	Dr. Erin Michalak is a Professor in the Department of Psychiatry at the University of British Columbia in Vancouver, Canada. She is a world leader in consumer-involved psychosocial research in bipolar disorders (BD). Dr. Michalak founded and leads the Collaborative RESearch Team to study psychosocial factors in Bipolar Disorder			

(CREST.BD). CREST.BD's mandate is to advance research, clinical practice, and social change for psychosocial care and supports for BD through a community-based participatory research (CBPR) approach. Regionally, she is the BC SUPPORT Unit's Patient Engagement Methods Cluster Lead (part of the Canadian Strategy for Patient Oriented Research), overseeing a budget of one million dollars to advance patient-engaged research in BC. Additionally, she is the Program Director for the APEC Digital Hub for Mental Health and VP for Outreach for the International Society for Bipolar Disorders.

Dr. Michalak's research expertise lies in mood disorders, digital mental health, patient engagement in research, knowledge translation (KT), quality of life (QoL), and global mental health. She has published over 150 scientific articles and several books and book chapters. She has been awarded the 2018 CIHR Gold Leaf Prize for Transformation in Patient Engagement, Canada's most prestigious recognition for patient engagement in research across all health disciplines. She has also been awarded the 2019 Douglas Utting Prize, made annually for significant accomplishments in promoting the awareness and treatment of depression in Canada. Dr. Michalak has experience with developing inter-governmental white papers and reports as part of her leadership role with the APEC Digital Hub for Mental Health (for example, she co-led the consultation activities for and co-wrote 'A New Horizon for Occupational Health: APEC White Paper on Workplace Mental Health and Safety'. Dr. Michalak also sits on the Scientific Advisory Board of DBSA and was a key academic expert in their program of work to advance understandings of wellness in people with BD.

Dr. Emma Morton is a Senior Lecturer (Teaching and Research) in the School of Psychological Sciences at Monash University. She completed her PhD and clinical training as a psychologist at Swinburne University of Technology, Australia and her postdoctoral research fellowship at the University of British Columbia. Dr. Morton is now the co-director of the Collaborative Research Team to Study Psychosocial Issues in Bipolar Disorder (CREST.BD). Her research experience spans psychosocial and quality of life-oriented treatments for bipolar disorder, development and administration of digital mental health interventions and optimizing the measurement and treatment of outcomes valued by patients living with mood disorders using a community-based participatory research framework. Dr. Morton is a 2021-2022 Canadian Institutes of Health Research Banting Postdoctoral Fellow. She was named the recipient of the 2021 Depression and Bipolar Support Alliance Gerald L. Klerman Young Investigator Award, and the 2022 International Society of Bipolar Disorders Samuel Gershon Junior Investigator Award.

Andrew Kcomt is the Knowledge Translation & Research Manager

	at Hope & Me - Mood Disorders Association of Ontario. As the co-chair of the CAN-BIND Community Advisory Committee, he represents the community perspective on the Ontario Brain Institute's Knowledge Translation, Outreach, and Communications team and has been a long-time community advisor with Collaborative RESearch Team to study psychosocial issues in Bipolar Disorder (CREST.BD) and the Centre For Youth Bipolar at CAMH. His interests include patient advocacy, team building, diversity enhancement, and collaboration.<br><br>Conflict of Interest Statement: We confirm that none of the study team members will have relationships with program participants/ facilitators. Study team members will not be in a position of authority over a participant/facilitator. If any conflict of interest is perceived or arises, the study team member will absolve themselves from participating in recruitment or data collection activities.
**8. Confidentiality - Behavioural Study** [View Form]	
8.1. Security of Data During the Course of the Study	All information and data collected will be password-protected and encrypted, and will be accessed only using university-encrypted and owned computers. Audio/video recorded interviews will be transcribed. Electronic data files will be stored on UBC's secure OneDrive server. Any data shared with Co-Investigators will be done through UBC OneDrive and will be de-identified and password-protected. Interview recordings will be transcribed by a transcription service. Audio/video recorded interviews conducted using Zoom will be downloaded locally to the interviewer's computer and immediately transferred to a secure UBC server. Pyschoeducation program sessions will not be recorded, nor will any participant-completed activities (e.g., worksheets, etc.) be collected. Information collected by Hope+Me for their standard operating procedures (e.g., program registration forms and group peer support summaries) will be password-protected and encrypted, and will be accessed only using Hope+Me owned computers by approved staff.<br><br>All research questionnaires (surveys, consent form, demographic questions) will be administered via Qualtrics using our university Qualtrics account. Qualtrics is designed to gather sensitive questionnaire data online, and it incorporates a number of procedures for minimizing risk of a breach of confidentiality. We will set up our data collection so that IP addresses are not stored. Qualtrics servers are protected by high-end firewall systems, and vulnerability scans are performed regularly. Complete penetration tests are performed yearly. All services have quick failover points and redundant hardware, and complete backups are performed nightly. Our electronic dataset will be de-identified, encrypted, password protected, and will only be accessible by core research team members. The electronic data will be kept on password-protected accounts and will be transferred to a limited number of

	university-owned and -maintained computers of Dr. Erin Michalak. No research data will be transferred to Hope+Me.
8.2. Access to Data	The UBC study team and research staff will have ready access to the data. A transcription service located in Canada will be given access to de-identified audio files only for the purposes of working on interview transcriptions.
8.3. Protection of Personal Information	Program evaluation surveys completed by facilitators and participants will not collect any personally identifying information (e.g., names, contacts, etc.). Data from surveys will not be made available to facilitators and participants during the research study to minimize bias in the results.<br><br>We will record audio (and, if participants join via the Zoom videoconferencing platform, video) data of the (optional) qualitative interview, however this will not be linked or stored with identifiable data.<br><br>Any personally identifying information (e.g., name, postal code and emergency contact information) collected by Hope+Me to register for the program is strictly part of standard operating procedures and will only be used in the event of an emergency. All identifying information will only be accessible to the on-call Hope+Me staff member providing one-on-one support for program activities, and will be destroyed upon completion of the program.<br><br>We have three major types of identifiable data: (a) contact information (names, email address, and phone number), (b) consent forms and (c) demographics and clinical information. Each of these data files will be stored separately from research data, only being linked through a subject ID number - the file containing this list will be password protected, encrypted and stored separately from the data.<br><br>Participant data will be kept confidential, except for certain information that we must report, such as abuse of a child, to the RCMP as part of our professional duty. Additionally, if the research team has concerns about participant responses to survey or interview questions, we reserve the right to reach out to Hope+Me to enquire about their participation in the program.
8.4. Transfer of Data<br><br>*Will any data be transferred (made available) to persons or agencies outside the lead University or Health Authority?*	yes

If yes, describe in detail what information will be released, to whom, how the data will be transferred, how and where it will be stored and what safeguards will be used to protect the identity of participants and the privacy of their data. Attach the data transfer agreement if applicable.	Co-investigator Dr. Emma Morton is based at Monash University, in Melbourne, Australia. Data will be shared through UBC OneDrive and will be de-identified and password-protected. If a copy of the data will need to be stored on a local computer for analysis, the data will be stored through Monash Network Storage, managed by eSolutions and stored on servers owned by Monash University. All data on these servers is automatically backed up by the University, requires a VPN to access when off-site, has restricted access options and is encrypted.<br><br>Additional information about Monash University's data security protocols can be found through this link:<br><br>https://www.monash.edu/library/researchers/data-collection-management/guidelines/storage
8.5. Retention and Destruction of Data	Identifying information will be deleted at the end of the research study. Digital files containing de-identified, anonymized data will be retained for 5 years after study completion. Data will be stored at UBC and the PI will be responsible for this data.
8.6. Future Use of Data	Summarized results from this study may be published in academic journals, presented at conferences, and/or used for educational purposes. No study participants will be identified by name in any reports of the completed study.
8.7. Feedback to Participants<br><br>*Please provide information regarding your plans for communicating study results to participants. See the guidance notes for more information and respond to the bullet points as needed.*	Results of this study are not expected to be personally meaningful to individual participants; therefore personal contacts with study findings will not be made. However, findings and progress updates relative to the study will be communicated to participants as the project proceeds. We will blog regularly about study progress and findings on our CREST.BD research blog.

9. Documentation - Behavioural Study [View Form]

	Document Name	Version	Date	Password (if applicable)	
9.1. Research Proposal	Research Protocol	2	May 7, 2025		[View]
	Grant Application	1	November 25, 2024		[View]
	Research Protocol	1	November 25, 2024		[View]

9.2. Documentation of Consent	**Document Name** / **Version** / **Date** / **Password (if applicable)**

Document Name	Version	Date	Password (if applicable)	
Informed Consent - Facilitators	3	May 7, 2025		[View]
Informed Consent - Attendees	3	February 14, 2025		[View]
Informed Consent - Facilitators	3	February 14, 2025		[View]

9.3. Documentation of Assent

Document Name	Version	Date	Password (if applicable)

9.4. Advertisement to Recruit Participants *(Ads, Posters, letters of initial contact, etc).*

Document Name	Version	Date	Password (if applicable)	
Social Media Study Recruitment Posts - Attendees + Facilitators	3	May 7, 2025		[View]
Recruitment Email for Attendees + Facilitators	3	May 7, 2025		[View]
Recruitment Email for Attendees + Facilitators	2	January 16, 2025		[View]
Social Media Study Recruitment Posts - Attendees + Facilitators	2	January 16, 2025		[View]

9.5. Questionnaire, Questionnaire Consent Cover Letter, Tests, Interview Scripts, etc.

Document Name	Version	Date	Password (if applicable)	
Facilitator Demographics	3	May 7, 2025		[View]
Attendee Followup Survey	2	February 14, 2025		[View]
Qualitative Interview Script - Attendee	3	February 14, 2025		[View]
Qualitative Interview Script - Facilitator	3	February 14, 2025		[View]
Attendee Post Survey	2	February 14, 2025		[View]
Weekly Facilitator Feedback	2	February 14, 2025		[View]
Attendee Demographics	2	January 16, 2025		[View]
Facilitator Demographics	2	January 16, 2025		[View]
Hope+Me Program Registration Form	1	January 16, 2025		[View]

	Attendee Pre Survey	1	November 21, 2024	[View]
9.6. Letter of Initial Contact	**Document Name**	**Version**	**Date**	**Password (if applicable)**

9.7. Other Documents	**Document Name**	**Version**	**Date**	**Password (if applicable)**
	Letter of Support	1	December 20, 2025	[View]
	Is Peer Support the Right Path for You	1	January 16, 2025	[View]
	Expressing Needs and Requesting Support for Facilitators	1	January 16, 2025	[View]
	Group Peer Support Summary	1	January 16, 2025	[View]
	Guidelines for Virtual Engagement	1	January 16, 2025	[View]
	Reflective Practice Model for Training and Group Facilitators	1	January 16, 2025	[View]
	Risk and Distress Protocol	2	January 16, 2025	[View]
	Drop-In Support Case Notes	1	January 16, 2025	[View]
	Response Quality and Bot Checking Protocol	1	January 16, 2025	[View]
	Peer Facilitator Guidelines and Supports	1	January 16, 2025	[View]
	Facilitator Manual	1	November 25, 2024	[View]
	Attendee Manual	1	November 25, 2024	[View]

9.8. Websites and Social Media	Social Media:<br><br>X - https://x.com/CREST_BD<br>Instagram - https://www.instagram.com/crest.bd/?hl=en<br>Facebook - https://www.facebook.com/CRESTBD/<br><br>Website:<br><br>https://www.crestbd.ca/

10. Fee for Service - Behavioural Study [View Form]

How to submit<br><br>*Please indicate which of the following methods of payment will be used for this application:*	N/A (Not funded by an Industry For-Profit sponsor)
Please wait for the invoice from the UBC Behavioural Research Ethics Board (BREB) to submit payment. The invoice will detail payment instructions and wire transfer information.<br><br>***Contact information regarding where to send the invoice.***	Caden Poh:<br>caden.poh@ubc.ca<br><br>Please CC the following emails:<br>crest.bd@ubc.ca<br>sahil.kanani@ubc.ca

12. Save Application - Human Ethics [View Form]

Print Close
