# Supplementary material for: ‘Thriving with bipolar disorder’: The co-design of a peer-delivered group psychoeducation program and single-arm pilot feasibility evaluation protocol
Source: PLoS One. 2025 Dec 17;20(12):e0338306. doi: 10.1371/journal.pone.0338306 (PMC12711008; doi:10.1371/journal.pone.0338306)
Supplement: S3 File — Semi-structured qualitative interview guides to be used with attendees and facilitators. (DOCX) [file pone.0338306.s003.docx]

# S3 File. Semi-structured interview guides

## Qualitative Interview Guide (Attendee)

**Introduction:**

Hello ___, this is Emma Morton from Monash University in regards to the “Thriving with bipolar disorder” group program. Is now still a good time for our chat?

The focus of this interview is to learn about your experiences - good or bad - of participating in the “Thriving with bipolar disorder” group program. In the first set of questions I will ask you about you for your feedback on the program. In the second set of questions I will ask you what you learned through the program, and whether you made any changes in your life as a result of what you learned. The final set of questions will be about how you feel about managing your bipolar disorder right now.

At the end of the interview, you’ll have time to add any of your thoughts that you think are important to our research but that weren’t discussed. The interview will take up to an hour.

**Re-Consent:**

Before we commence with the interview, we would like to remind you that your participation in this study is entirely voluntary. You may withdraw from this study at any time by informing me, or a research team member via email at [crest.bd@ubc.ca](mailto:crest.bd@ubc.ca).

Withdrawing from the study, or your level of participation in this program, will not result in the loss of any of your benefits or rights, nor will it impact your employment, any medical care you might receive in the future, or your participation in any other research studies/activities. Additionally, you will be offered the honorarium for each study activity you participate in, even if you withdraw part way through the study activity or the program altogether.

If you have any questions about the study, please feel free to ask me, or contact the research team via email at [crest.bd@ubc.ca](mailto:crest.bd@ubc.ca). If you have any concerns or complaints about your rights as a research participant and/or your experiences while participating in this study, contact the Research Participant Complaint Line in the UBC Office of Research Ethics (phone: 604-822-8598, phone (toll-free): 1-877-822-8598, email: [rsil@ors.ubc.ca](mailto:rsil@ors.ubc.ca)). Please note that this office is not part of the research team – Everything that you discuss will be kept confidential. The Ethics ID number for this study is H24-03489.

As you know, I’ll be recording this interview, as we want to be sure we accurately capture everything you have to say. After we transcribe the interview, the audio file will be deleted, and your name and identifiers, like your location, will be removed during transcription. We will not identify anyone by name in our findings, and all your data will be kept confidential and de-identified. We may publish anonymized quotes to ensure we represent your experiences faithfully. If you feel uncomfortable and don’t want to answer a question or would like to stop the interview, you are welcome to do so at any time. After the interview, you'll receive a gift certificate to thank you for your time.

Do you have any questions before we begin?

Do I have your consent to proceed with this interview?

I’m going to start recording now.

**First set: Intervention feedback**

How did you hear about the program and what made you decide to take part?

What was the most helpful part of the program?

Was there anything about the program that was difficult or challenging?

What was it like to participate in a program facilitated by a peer with their own lived experience?

- Probe if not discussed: Was there anything the facilitator did that was particularly helpful or unhelpful?

What was it like to interact with other people in the groups?

- Probe if not discussed: Was there anything the group members did that was particularly helpful or unhelpful?

If you could change or add anything to this program, what would it be?

- Probe for: feedback on program content, delivery, the facilitator, and the group environment

**Second set: Behaviour change**

Did you learn anything new from participating in this program?

- Probe: If yes, what did you learn?
- Probe: If no, why is that?

Did you make any changes in your life as a result of what you learned from the program?

- Probe: If yes, how so?
- Probe: If no, why is that?

**Third set: Impacts**

Since participating in the program, have you felt any different in terms of your ability to manage any difficulties related to your bipolar disorder?

- Probe: If yes, what has changed?
- Probe: If no, why is that?

Since participating in this program, have you noticed any changes in how you feel about yourself or your quality of life?

- Probe: If yes, what has changed?
- Probe: If no, why is that?

**Conclusion:**

Thank you for all of your feedback on the program so far. I just have one more question for you.

Would you recommend this program to someone else living with bipolar disorder?

- Probe: If yes, why?
- Probe: If no, why is that?

That’s all the questions that I have for you today. Is there anything else you would like to share with me or comment on?

*End with a thank you for their time and sharing their insights about their experiences.*

## Qualitative Interview Guide (Facilitator)

**Introduction:**

Hello ___, this is Emma Morton from Monash University in regards to the “Thriving with bipolar disorder” group program. Is now still a good time for our chat?

The focus of this interview is to learn about your experiences - good or bad - of facilitating the “Thriving with bipolar disorder” group program. In the first set of questions I will ask you about your feedback about the training and support you received when facilitating this program. In the second set of questions I will ask you for your feedback on the program content and format, and your observations about what worked or didn’t work well. The final set of questions will be about what the experience of being a facilitator was like for you personally.

At the end of the interview, you’ll have time to add any of your thoughts that you think are important to our research but that weren’t discussed. The interview will take up to an hour.

**Re-Consent:**

Before we commence with the interview, we would like to remind you that your participation in this study is entirely voluntary. You may withdraw from this study at any time by informing me, or a research team member via email at [crest.bd@ubc.ca](mailto:crest.bd@ubc.ca).

Withdrawing from the study, or your level of participation in this program, will not result in the loss of any of your benefits or rights, nor will it impact your employment, any medical care you might receive in the future, or your participation in any other research studies/activities. Additionally, you will be offered the honorarium for each study activity you participate in, even if you withdraw part way through the study activity or the program altogether.

If you have any questions about the study, please feel free to ask me, or contact the research team via email at [crest.bd@ubc.ca](mailto:crest.bd@ubc.ca). If you have any concerns or complaints about your rights as a research participant and/or your experiences while participating in this study, contact the Research Participant Complaint Line in the UBC Office of Research Ethics (phone: 604-822-8598, phone (toll-free): 1-877-822-8598, email: [rsil@ors.ubc.ca](mailto:rsil@ors.ubc.ca)). Please note that this office is not part of the research team – Everything that you discuss will be kept confidential. The Ethics ID number for this study is H24-03489.

As you know, I’ll be recording this interview, as we want to be sure we accurately capture everything you have to say. After we transcribe the interview, the audio file will be deleted, and your name and identifiers, like your location, will be removed during transcription. We will not identify anyone by name in our findings, and all your data will be kept confidential and de-identified. We may publish anonymized quotes to ensure we represent your experiences faithfully. If you feel uncomfortable and don’t want to answer a question or would like to stop the interview, you are welcome to do so at any time. After the interview, you'll receive a gift certificate to thank you for your time.

Do you have any questions before we begin?

Do I have your consent to proceed with this interview?

I’m going to start recording now.

**First set: Training and support**

Why did you decide to join as facilitator of the ‘Thriving with bipolar disorder’ program?

Was the training that you received to deliver the program useful?

- Probe if yes: What did you find the most helpful?
- Probe if no: How do you think the training could be improved?

What were your impressions of the facilitator manual?

- Probe: Was the manual useful for you when preparing for and running the sessions?
- Probe: Are there materials other than the manual that you think would be useful for facilitators?

**Second set: Experiences of program facilitation**

Thinking now about your experiences of facilitating the ‘Thriving with bipolar disorder’ program, can you tell me your thoughts about the topics and strategies covered?

- Probe if not discussed: How well did participants engage in the discussion and activities?
- Probe if not discussed: Adherence to the manual and reasons for non-fidelity

Did anything stand out to you about how the group members interacted with one another?

- Probe: What helped the groups run well?
- Probe: Was there anything that was difficult or challenging in your groups?

What aspects of the ‘Thriving with bipolar disorder’ program were most helpful? Least helpful?

If you could change or add anything to this program, what would it be?

- Probe for: feedback on program content, delivery, the facilitator role, and the group environment

**Third set: Personal changes and experiences of peer support facilitation**

Were you able to share your own lived experience in your role as facilitator?

- Probe if yes: What was that like for you? How did participants react to this information?
- Probe if no: Why was that?

Has facilitating this program helped you in your own self-management in any way?

- Probe: If yes, how so?

**Conclusion:**

Thank you for all of your feedback on the program so far. I just have one more question for you.

Would you recommend this program to someone else living with bipolar disorder?

- Probe: If yes, why?
- Probe: If no, why is that?

That’s all the questions that I have for you today. Is there anything else you would like to share with me or comment on?

*End with a thank you for their time and sharing their insights about their experiences.*
